# Isolation and Characterization of Methicillin-Resistant *Staphylococcus aureus* from Pork Farms and Visiting Veterinary Students

**DOI:** 10.1371/journal.pone.0053738

**Published:** 2013-01-03

**Authors:** Timothy S. Frana, Aleigh R. Beahm, Blake M. Hanson, Joann M. Kinyon, Lori L. Layman, Locke A. Karriker, Alejandro Ramirez, Tara C. Smith

**Affiliations:** 1 Department of Veterinary Diagnostic and Production Animal Medicine, College of Veterinary Medicine, Iowa State University, Ames, Iowa, United States of America; 2 University of Iowa, Center for Emerging Infectious Diseases, Department of Epidemiology, College of Public Health, Iowa City, Iowa, United States of America; University of Edinburgh, United Kingdom

## Abstract

In the last decade livestock-associated methicillin-resistant *S. aureus* (LA-MRSA) has become a public health concern in many parts of the world. Sequence type 398 (ST398) has been the most commonly reported type of LA-MRSA. While many studies have focused on long-term exposure experienced by swine workers, this study focuses on short-term exposures experienced by veterinary students conducting diagnostic investigations. The objectives were to assess the rate of MRSA acquisition and longevity of carriage in students exposed to pork farms and characterize the recovered MRSA isolates. Student nasal swabs were collected immediately before and after farm visits. Pig nasal swabs and environmental sponge samples were also collected. MRSA isolates were identified biochemically and molecularly including *spa* typing and antimicrobial susceptibility testing. Thirty (30) veterinary students were enrolled and 40 pork farms were visited. MRSA was detected in 30% of the pork farms and in 22% of the students following an exposure to a MRSA-positive pork farm. All students found to be MRSA-positive initially following farm visit were negative for MRSA within 24 hours post visit. Most common *spa* types recovered were t002 (79%), t034 (16%) and t548 (4%). *Spa* types found in pork farms closely matched those recovered from students with few exceptions. Resistance levels to antimicrobials varied, but resistance was most commonly seen for spectinomycin, tetracyclines and neomycin. Non-ST398 MRSA isolates were more likely to be resistant to florfenicol and neomycin as well as more likely to be multidrug resistant compared to ST398 MRSA isolates. These findings indicate that MRSA can be recovered from persons visiting contaminated farms. However, the duration of carriage was very brief and most likely represents contamination of nasal passages rather than biological colonization. The most common *spa* types found in this study were associated with ST5 and expands the range of livestock-associated MRSA types.

## Introduction

*Staphylococcus aureus* is a common bacterium found on the skin and nasal passages of healthy people. Approximately 25–40% of the population is colonized with *S. aureus*. It is also a common cause of skin and soft tissue infections and sometimes causes severe disease such as pneumonia, bacteremia, meningitis, sepsis, and pericarditis. *S. aureus* bacteria harboring the *mecA* gene are resistant to methicillin and other β-lactam antimicrobials and are referred to as methicillin-resistant *S. aureus* (MRSA). In the United States it is estimated that 1.5% of the population (∼4.1 million persons) is colonized with MRSA [1] leading to at least 94,000 invasive infections and over 18,000 deaths annually [2]. Various categories of MRSA based on epidemiologic characteristics are commonly used and include healthcare-associated MRSA (HA-MRSA), community-associated MRSA (CA-MRSA) and livestock-associated MRSA (LA-MRSA). HA-MRSA infections are most commonly found in immunocompromised people who have spent time in hospitals or healthcare centers, while CA-MRSA infections occur among otherwise healthy adults and children in the wider community. Livestock-associated MRSA (LA-MRSA) refers to strains of MRSA in which animals, particularly production animals, serve as the main reservoir of infection to humans.

LA-MRSA emerged as a public health concern in 2005 with reports of a specific multilocus sequence type (ST398) being found in higher than expected numbers in swine workers in France and the Netherlands [3]–[5]. Since ST398 was found at high levels in both pigs and pig farmers and very low levels in the general population, it was initially referred to as the “swine-associated” MRSA. Several studies attempting to determine the prevalence of ST398 in pigs have been conducted including a large multi-national study conducted by the European Food Safety Authority (EFSA) which found the prevalence of MRSA ST398 in swine farms to be 25.5% but varied from 0% to 50.2% among European Union Member States [6]. In Ontario, Canada a study found that 25% of the pigs from 20 farms were colonized with MRSA and that ST398 was the predominant sequence type [7]. A study in the U. S. examined 299 animals from two swine production systems in Iowa and Illinois and 45% were found to carry MRSA. All isolates typed were ST398 [8].

It is apparent that those workers who spend considerable time in production animal farms are more likely to carry MRSA than those who don't. One study in The Netherlands demonstrated a 26% carriage rate among pig farmers [4]. The Canadian and U. S. studies previously mentioned found MRSA is 20% and 45%, respectively, in the swine workers tested. Isolates obtained from swine and their human caretakers are frequently indistinguishable, suggesting transmission between the two animal species [7]. Several studies have indicated that veterinarians working with swine are more likely to carry MRSA, primarily ST398, than non-swine focused colleagues [9]–[12]. While there are concerns that ST398 may establish itself in people, it appears that human to human spread of ST398 is limited [13], [14] and transmissibility within hospitals is less likely than non-ST398 MRSA strains [15], [16]. Additionally, colonization in persons exposed to livestock appears to be dependent on intensity of animal contact [17]. Studies indicate that short-term exposure to MRSA-positive pig farms does not lead to long-term colonization [17], [18]. Similar studies assessing the risk of short but intense exposure to MRSA-positive pork farms in the U. S. have not been done. Therefore the objectives of this study were to: i) assess the rate of MRSA acquisition and longevity of carriage in uncolonized students exposed to pork farms during the two week course, ii) characterize recovered MRSA isolates by *spa* typing and antimicrobial susceptibility testing to assess the relatedness between pork farms and veterinary student isolates.

## Methods

### Ethics Statement

The ISU Institutional Review Board (IRB) approved the protocols. Animal samples tested were obtained from samples submitted as part of the diagnostic workup for field case investigations and did not require institutional animal care committee (IACUC) approval. All animals sampled were under a valid veterinary-client-patient relationship (VCPR).

### Enrollment

Veterinary students were provided written informed consent and voluntarily enrolled during participation in swine courses at Iowa State University (ISU) from May to November, 2010. Students answered a short questionnaire related to potential risk factors for MRSA such as recent respiratory illness with fever and sore throat, skin or soft tissue infections (SSTI), antibiotic use, hospitalization, visitation to pork production or prior diagnosis of MRSA. Age and gender information was also collected. Students participated in diagnostic investigations at pork farms as would normally occur during the two-week clinical swine medicine fourth year elective course. Diagnostic investigations at pork farms were based on requests to ISU Veterinary Diagnostic and Production Animal Medicine (VDPAM) department by swine veterinarians and producers seeking assistance with animal health-related problems. Students were randomly assigned to an investigation and were generally at the pork farms for 3 to 4 hours. No prior knowledge of MRSA status or MRSA-related disease in pigs or humans at the pork farms was available. The type of farm and approximate age of animals were recorded at the time of visit, but no further farm data was made available for this study.

### Sample collection

#### Student

Students were sampled at the following intervals: 1) the beginning of the course before any visits to pork farms, 2) before entry into a pork farm, 3) immediately after leaving a pork farm, 4) weekends or non-visit weekdays during the course, 5) daily for 4 consecutive days after the end of the clinical swine medicine course. Sample collection was accomplished by using sterile swabs (BBL CultureSwab, Sparks, MD) containing Stuart's medium inserted approximately 2 cm into one naris, rotated against the anterior nasal mucosa and repeated with same swab in second naris. The swabs were transported on ice to the ISU Veterinary Diagnostic Laboratory (VDL) within 6 hours. All samples were submitted using an assigned student study ID and date.

#### Animal

As part of the routine diagnostic investigation, when nasal samples were collected from manually restrained pigs for other diagnostic purposes, 3–5 of these nasal samples where then also submitted for MRSA testing. All samples were obtained as part of normal diagnostic investigation during student visit using materials and techniques described above for students. Samples were identified using a sample kit ID and date. Pigs were selected from pens with and without illness. Health status of the pig was not included when the sample was forwarded for MRSA testing.

#### Environmental

The environmental samples were collected from the same farms visited by participating students during the time of the visit. The sampling sites included, but were not limited to, treatment carts, fences and gates. Typically swab samples were collected from 3–5 areas in each farm. Samples were acquired by swabbing an approximate three square inch area with a sterile Speci-Sponge (Nasco, Fort Atkinson, WI) in 5 ml of enrichment broth, placed in Whirlpak bag, and transported on ice to the ISU VDL within 6 hours. Samples were identified using the date and same sample kit ID used for animal samples.

To maintain client confidentiality, each farm was assigned a farm study ID by an individual not involved in the study. A master spreadsheet was created that included the farm ID, sample kit ID, student IDs that visited the farm, sampling date, farm type, and approximate pig age.

### Isolation and identification of bacteria

Student and pig nasal swabs were inoculated directly into 2 ml of enrichment broth containing 10 g tryptone/L, 75 g NaCl/L, 10 g mannitol/L and 2.5 g yeast extract/L. Bags containing environmental sponges received an additional 10 ml of enrichment broth. Samples were incubated for 24 h at 35°C, then inoculated onto selective MRSA agar plates (MRSASelect, Bio-Rad, Hercules, CA), which were then incubated for 24–48 hours at 35°C. All plates were examined for MRSA and *Staphylococcus* species. Up to 3 suspect colonies from each sample were further identified by biochemical tests (coagulase, maltose, lactose, trehalose, and Voges-Proskauer). All *S. aueus* isolates were screened for methicillin resistance by disc diffusion (6 μg/ml oxacillin) on Mueller Hinton agar with 2% NaCL. Oxacillin-resistant isolates were tested for the presence of penicillin binding protein 2′ (PBP 2a) using latex agglutination kit (MRSA latex agglutination test, Oxoid Ltd., Hants, UK). At least one *S. aureus* isolate which was also PBP 2a positive from given sample was forwarded for molecular testing.

### Molecular testing

Genomic DNA was extracted using the Wizard Genomic DNA preparation kit (Promega, Madison, WI). Polymerase Chain Reaction (PCR) was performed on all isolates. A multiplex PCR assay was used to determine the presence of the *mecA* gene, and the *nuc* gene (present only in *S. aureus*) [19]). Amplification of the *Staphylococcus* protein A (*spa*) gene was performed through PCR as previously described [20], using primers validated for use with Ridom-StaphType software [21]. The presence of PVL toxin genes (*lukS, lukF*) was determined by an additional PCR [22]. All molecular procedures utilized known positive and negative controls.

### Antimicrobial Susceptibility Testing

Isolates were selected for antimicrobial susceptibility testing by broth dilution using minimum inhibitory concentration (MIC) method as described by the Clinical and Laboratory Standards Institute [23] using TREK Veterinary Sensititre equipment (Thermo Fisher Scientific, Cleveland, OH). Isolates were tested for susceptibility to chlortetracycline (CHL), clindamycin (CLI), enrofloxacin (ENR), florfenicol (FLO), gentamicin (GEN), neomycin (NEO), oxytetracycline (OXY), spectinomycin (SPE), sulfadimethoxine (SUL), tiamulin (TIA), tilmicosin, (TIL) and trimethoprim/sulfamethoxazole (TMP/SMZ). Beta-lactam antimicrobials were not considered. Breakpoints used for interpretation of resistance were based on information provided by TREK Diagnostic Systems and were as follows: CHL (≥8 µg/ml), CLI (≥2 µg/ml), ENR (≥1 µg/ml), FLO (≥4 µg/ml), GEN (≥8 µg/ml), NEO (≥8 µg/ml), OXY (≥8 µg/ml), SPE (≥32 µg/ml), TIA (≥32 µg/ml), TIL (≥16 µg/ml), TMP/SMZ (≥2 µg/ml). Multidrug resistance was defined as resistance to ≥4 antimicrobials. The reference strain *S. aureus* ATCC 29213 served as a quality control strain in the MIC determinations.

### Data Analysis

Descriptive analyses were initially performed. Factor associations were investigated using χ^2^ analysis and assessed with Fisher's exact test. Associations were deemed significant at *p*<0.05 level and subsequently odd ratios (OR) determined as appropriate. No allowance was made for multiple comparisons. Statistical analysis of data sets was performed using SAS software, version 9.1 (SAS Institute, Inc., Cary, NC).

## Results

### Pork farms samples

Forty (40) pork farms of various types and animal age groups were visited during the study period. No farm was visited more than once. MRSA was detected in 30% (12/40) of the pork farms tested by either pig or environmental sampling. Two sites did not have pig samples collected, but were positive for MRSA from the environmental samples. A total of 362 samples were collected from these sites including 194 from pigs and 168 from the environment. Overall MRSA was detected in 17.4% (63/362) of the samples tested including 17.5% (34/194) of the pig samples and 17.3% (29/168) of the environmental samples. In MRSA-positive farms, either animal or environmental samples were positive 60.1% (63/104) of the time. Of these, 69.4% (34/49) of pig samples and 52.7% (29/55) of environmental samples were MRSA-positive. There was no significant differences in MRSA detection between pig and environmental samples (*p = *0.08). Pig and environmental sample results at the farm level matched 97.4% (37/38) of the time. The type of farm and age of animals was recorded for 82.5% (33/40) farms visits. In MRSA-positive farms, pigs less than 10 weeks of age were nearly 6 times (OR 5.95; 95% CI 1.22–28.95) more likely to also be present than not. Pork farm sample testing results are summarized in Table 1.

**Table 1:** Overview of the characteristics for the pork farms visited in this study.

Facility Type	Age Range/Group	Pigs <10 weeks of age present	Number in study	Number with MRSA
Finisher	10–27 weeks	No	20	4
Farrow to finish	All age groups	Yes	3	0
Farrow to feeder	Birth – 10 weeks and Adults	Yes	5	5
Nursery	3–10 weeks	Yes	1	1
Sow Farm	Birth – 3 weeks and Adults	Yes	3	1
Gilt Developer	3–8 months	No	1	0
Unknown	NA*	NA*	7	1
**Total**			**40**	**12**

* NA  =  Not available.

### Student samples

Thirty (30) veterinary students were enrolled in a study. Only one student elected not to participate as she was taking the clinical swine course for a second time. Complete questionnaires were available for 29 students. The mean student age was 26.4 with a range of 24–35. Twenty females and 10 males participated in the study. Seven students reported using antibiotics in the previous 3 months. Also in previous 3 months, 0, 3, 1, 17 students reported hospitalization, respiratory disease with fever, SSTI, and pork farm visit, respectively. One student reported diagnosis of MRSA occurring 7 years prior. All students were negative for MRSA by nasal swab on the initial sampling. Six hundred and four (604) student samples were collected during the study period and MRSA was detected in 8 samples (1.3%, 8/604). Twenty-one (70%, 21/30) students visited MRSA-positive pork farms at least once and 6 students visited MRSA-positive farms on two separate occasions. Therefore, there were 27 student exposure events and MRSA was detected 6 times in separate students (22.2%, 6/27). MRSA was detected in 5 of these 6 students from the first nasal sample following the visit to a MRSA-positive farm. In one student MRSA was not detected until 5 days after a visit to a MRSA-positive farm. MRSA was not detected in any student for more than 24 hours, and no student subsequently became MRSA-positive again during the study period. MRSA was not detected in any student following visits to pork farms which were negative for MRSA. There was no significant association between detection of MRSA and recent respiratory disease with fever (*p = *0.53), recent antimicrobial use (*p = *0.29), SSTI (*p = *0.29), or recent swine farm visit (*p = *0.15). Additionally MRSA detection was not associated with gender (*p = *1.00) or multiple exposures to MRSA-positive farms (*p = *0.62). Age range in the exposed group was 24–35 years old. However, all except one student were between 24 and 28 years old. Therefore, age was not analyzed for risk. No students reported symptoms compatible with staphylococcal infections during the study period.

### Molecular testing

One hundred and six isolates from 69 separate samples were positive for both *mecA* and *nuc* genes and negative for PVL genes. All 106 MRSA isolates were *spa*-typed and results are shown in Table 2. In summary, six *spa* types were found including: t002 (78.3%; n = 83), t034 (14.2%; n = 15), t548 (4.7%; n = 5), t10065 (0.9%, n = 1), t126 (0.9%; n = 1), and t1107 (0.9%; n = 1). The *spa* types found in pork farms from either pig or environmental samples included: t002, t034, t548 and t10065. The *spa* types found in students included: t002, t034, t548, t1107, and t126. The sequence types (MLST) that have been associated with these *spa* types includes: ST398 (t034, t10065) [24], [25], ST5 (t002, t548, t1107) [21], [25], [26], and ST72 (t126) [21].

**Table 2:** Summary of the *spa* types and motifs from MRSA isolates found in this study overall and by source of isolation.

*Spa* type	Associated MLST	Motif	Overall	Pigs	Environment	Students
t002	ST5	26-23-17-34-17-20-17-12-17-16	83/106 (78.3%)	42/56 (75.0%)	31/37 (83.8%)	10/13 (76.9%)
t034	ST398	08-16-02-25-02-25-34-24-25	15/106 (14.2%)	10/56 (17.9%)	5/37 (13.5%)	-
t548	ST5	26-23-17-34-17-20-17-12-16	5/106 (4.7%)	4/56 (7.1%)	-	1/13 (7.7%)
t10065	ST398	02-16-12-25-02-25-34-24-25	1/106 (0.9%)	-	1/37 (2.7%)	-
t126	ST72	07-23-12-21-17-12-12-17	1/106 (0.9%)	-	-	1/13 (7.7%)
t1107	ST5	26-17-20-17-12-16	1/106 (0.9%)	-	-	1/13 (7.7%)

Pig and environmental *spa* types matched in all MRSA-positive farms with two exceptions. In one site, t034 was recovered from pig samples and one environmental sample. However, a second environmental sample from the same site was positive for MRSA with *spa* type t10065, which appears be a derivative of t034. In another site, t548 was recovered from all pig samples and t002 recovered from all environment samples. Both of these *spa* types (t548, t002) are associated with ST5 [25]. The *spa* type recovered from students and the pork farms closely matched those recovered from students with two exceptions; i) three *spa* types (t1107, t002, t548) were recovered from a student within 24 hours following exposure to a MRSA-positive farm where only t002 and t548 was detected. However, t1107 is also considered to be associated with ST5. ii) *spa* type t126, ST72-associated, was isolated from a student 5 days following exposure to a MRSA-positive farm with only *spa* type t002 detected. This isolate may represent exposure to a MRSA source not associated with pork farms. The combined results from pork farms and veterinary students are shown in Table 3.

**Table 3:** Combined results of environmental, pig, and veterinary student testing from MRSA-positive pork production sites.

Type of Facility	Pig Results^a^	Pig *spa* types	Environmental Results^a^	Environmental *spa* types	Student Results^b^	Student *spa* types
Finisher	NA		2/3	t002	0/1	
Finisher	NA		3/3	t002	0/1	
Sow Farm	4/5	t002	1/5	t002	3/3	t002; t126^c^
Nursery	1/5	t034	2/5	t034	0/3	
Finisher	5/5	t034	2/5	t034; t10065	0/3	
Finisher	2/5	t034	2/5	t034	0/2	
Farrow to Feeder	4/4	t002	2/4	t002	0/1	
Farrow to Feeder	5/5	t002	3/5	t002	1/3	t002
Farrow to Feeder	0/5		1/5	t002	0/3	
Farrow to Feeder	3/5	t002	2/5	t002	0/2	
Farrow to Feeder	5/5	t002	4/5	t002	1/3	t002
Unknown	5/5	t548	5/5	t002	1/2	t002; t548; t1107^d^
**Total**	**34/49**		**29/55**		**6/27**	

a Number of MRSA-positive samples/number of samples collected. ^b^Number of MRSA-positive students/number of students exposed. ^c^ *Spa* type t126 was isolated from a student 5 days following exposure to MRSA-positive site. ^d^Three *spa* types (t002, t548, t1107) from same student.

### Antimicrobial Susceptibility

**Figure 1:**
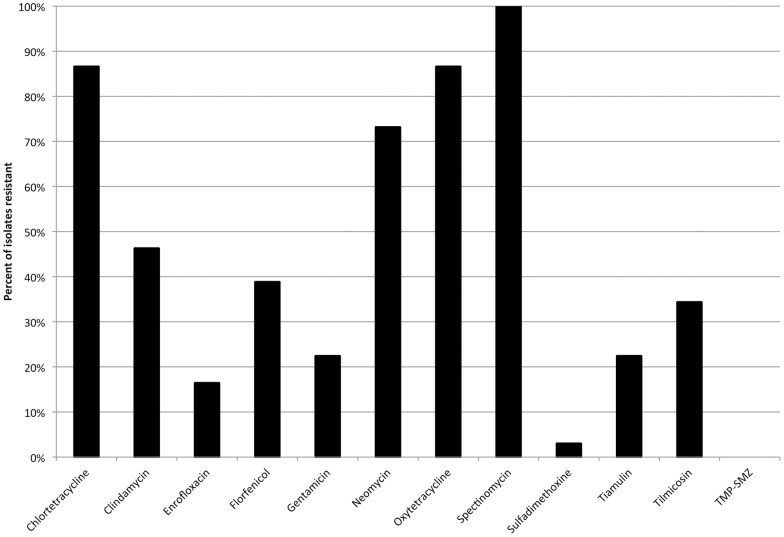
Antimicrobial resistance of MRSA isolates from pork farms and students. Results from 67 isolates tested.

**Figure 2:**
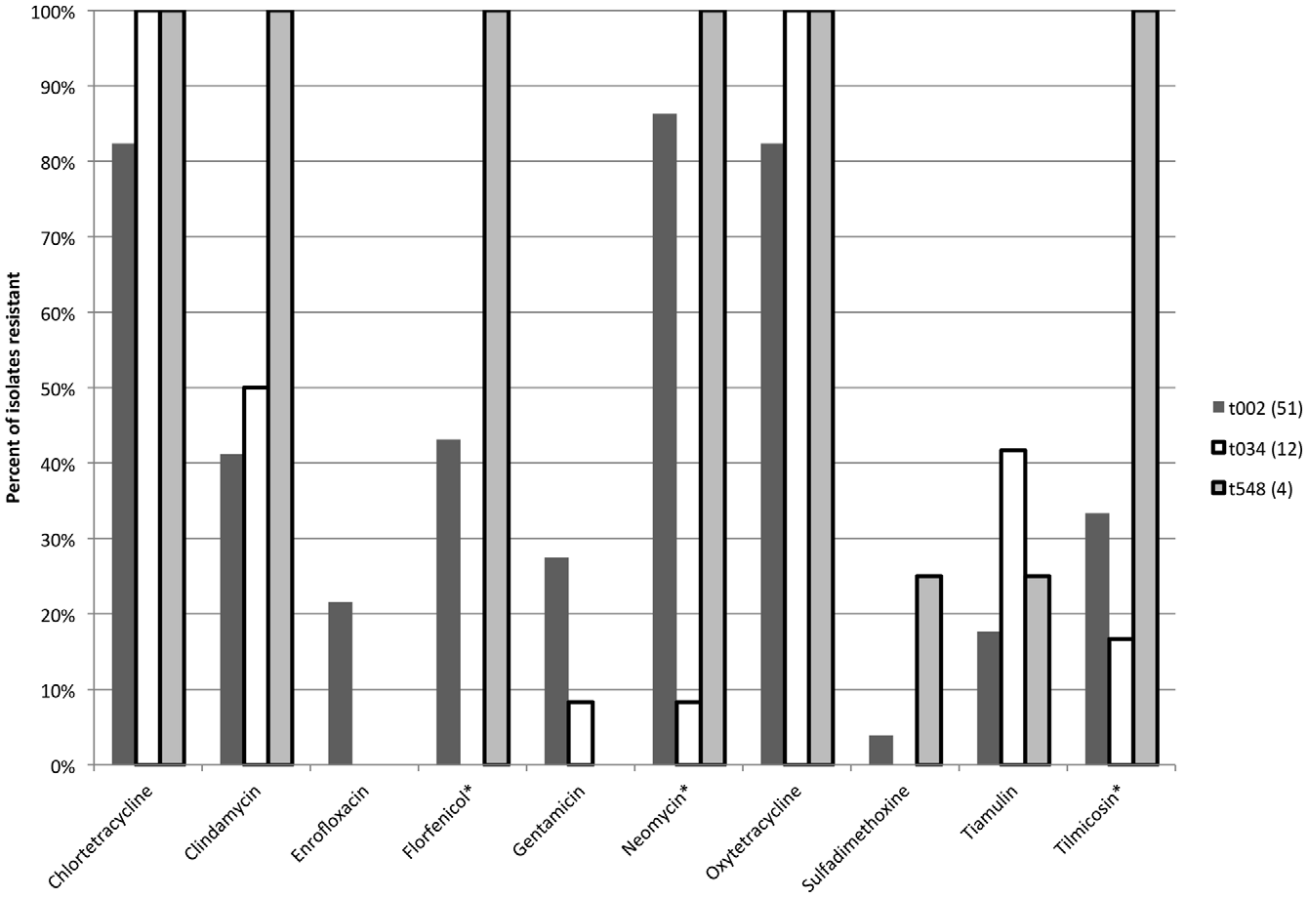
Antimicrobial resistance of MRSA isolates from pork farms and students. Number of isolates tested in parenthesis. Significantly different antimicrobial results across *spa* types indicated with asterisk (*).

**Figure 3:**
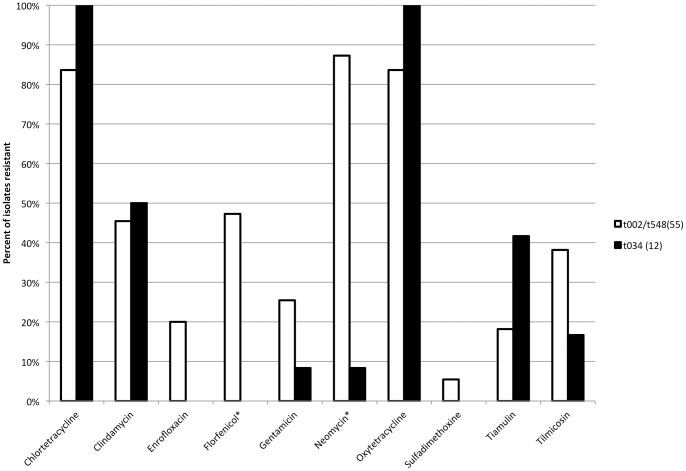
Antimicrobial resistance of MRSA isolates from pork farms and students by ST398 status. t034 considered ST398-associated and t002/t548 considered non-ST398-associated. Number of isolates tested in parenthesis. Significantly different antimicrobial results by *spa* types indicated with asterisk (*).

Antimicrobial susceptibility panel testing (AST) was performed on 67 MRSA isolates from separate samples. Sources of MRSA isolates for AST included: pigs (n = 31), environment (n = 28) and students (n = 8). The *spa* types for AST included: t002 (n = 51), t034 (n = 12) and t548 (n = 4). Resistant levels to antimicrobials for all isolates included: CHL (n = 58, 86.6%), CLI (n = 31, 46.3%), ENR (n = 11, 16.4%), FLO (n = 26, 38.8%), GEN (n = 15, 22.4%), NEO (n = 49, 73.1%), OXY (n = 58, 86.6%), SPE (n = 67, 100%), SUL (n = 2, 3.0%), TIA (n = 15, 22.4%), TIL (n = 23, 34.3%), TMP-SMZ (n = 0, 0.0%) Percentage of all isolates that were resistant to a given antimicrobial is shown in Figure 1. Significant differences in level of resistance by source were seen only with enrofloxacin (*p = *0.024) and florfenicol (*p = *0.0006). The student isolates were more resistant than farm isolates for both antimicrobials. Significant differences in level of antimicrobial resistance among *spa* types were seen for: FLO (*p = *0.0002), NEO (*p = *<0.0001), and TIL (*p = *0.01) as shown in Figure 2. When related *spa* types (t002, t548) were combined, significant differences compared to t034 were found for only FLO (*p = *0.002) and NEO (*p = *<0.0001) (Figure 3). In the case of NEO, if resistance was found the odds that the isolate was either t002 or t548 was very high (OR = 75.4, 95% CI = 8.4–677.6). There was 23 different resistant profiles in the isolates tested. The most common resistant phenotypes are shown in Table 4. Sixty -four (95.5%, 64/67) isolates were resistant to 3 or more antimicrobials. One isolate was resistant to 10 antimicrobials (t002; CHL-CLI-FLO-GEN-NEO-OXY-SPE-SUL-TIA-TIL). Combined resistance to tetracyclines (CHL, OXY), neomycin, and spectinomycin was seen in 67.2% (45/67) of the isolates overall but only in 8.3% (1/12) of the ST398 isolates. The proportion of multidrug-resistant isolates (≥4 antimicrobials) was higher in non-ST398 MRSA (94.5%, 52/55) versus ST398 (58.3%, 7/12) isolates (*p = *0.0005).

**Table 4:** Most prevalent antimicrobial resistant profiles found in MRSA isolates and associated *spa* types.

Resistance profile	No. isolates (%)	*spa* type(s) with pattern (#)
CHL-NEO-OXY-SPE	17/67 (25.4)	t002
CHL-CLI-FLO-NEO-OXY-SPE-TIL	10/67 (14.9)	t002 (7); t548 (3)
CHL-OXY-SPE	5/67 (7.5)	t034
CLI-ENR-FLO-GEN-NEO-SPE-TIL	3/67 (4.5)	t002
CHL-CLI-GEN-NEO-OXY-SPE-TIA	3/67 (4.5)	t002 (2); t034(1)
CHL-CLI-OXY-SPE-TIA	3/67 (4.5)	t034
CHL-FLO-NEO-OXY-SPE	3/67 (4.5)	t002

CHL  =  chlortetracycline, CLI  =  clindamycin, ENR  =  enrofloxacin, FLO  =  florfenicol, GEN  =  gentamicin, NEO  =  neomycin, OXY  =  oxytetracycline, SPE  =  spectinomycin, TIA  =  tiamulin, TIL  =  tilmicosin.

## Discussion

### MRSA transmission to students

In this study we investigated the transmission dynamics associated with MRSA found in pork farms. We found that following short-term exposure (3–4 hr) to MRSA-positive pork farms, MRSA could be detected in students approximately 22% of the time. However, MRSA was not detected in any students for more than one day post-farm visit and did not reappear later on in the study. This suggests that the strains of MRSA from the pork farms did not become established in the students. These findings are consistent with other studies investigating LA-MRSA that have shown that short-term exposure to production animal farms does not lead to colonization [18], [27] or that carriage rapidly decreases when exposure is removed [17]. Studies have investigated the prevalence of MRSA in occupationally exposed people such as veterinarians with varying results. Some studies have used convenience sampling conducted at meetings or conferences and found detectable MRSA in swine veterinarians at levels such as 3% [28], 3.9% [11], and 12.5% [29]. A cross-sectional study found the prevalence of MRSA in livestock veterinarians to be 1.4% and 9.5% in Denmark and Belgium, respectively [30], while an epidemiological study in Germany found 23% of meat inspectors, laboratory personnel, and veterinarians tested were positive for MRSA ST398 [12]. Differences in prevalence can be expected based on geographic location, frequency of exposure, time since exposure, veterinary practices and study design. However, the level of MRSA detection in students enrolled in this study is rather consistent with other veterinarian prevalence studies indicating that this study may accurately represent the occupational exposure encountered by swine veterinarians. Additionally this study might provide insight into possible transmission risk to other sectors of the population with limited animal contact, such as agricultural fairgoers or petting zoo visitors. An advantage of this study over point-in-time prevalence studies is that participants were sampled frequently over time and therefore represents true incidence and temporal association to exposure. Although certain risk factors were investigated in this study (i.e. recent respiratory illness, SSTI, antibiotic use, hospitalization, pork farm visit), sample size limits the extent to which any conclusions can be drawn regarding these risk factors. Future studies targeting known MRSA-positive pork farms would increase the level of exposure and allow better assessment of human risk factors and MRSA colonization, but this would require a different approach than what could be achieved with the limitations associated with this study.

### MRSA prevalence in pork farms

This study provides an estimate of the prevalence of MRSA on pork farms in the Midwestern U. S. While there have been a large number of studies examining prevalence of MRSA is pork farms in Europe [5], [6], [31]–[43], there have been rather few similar studies in the North America [7], [8]. However, finding MRSA in 30% of the pork farms in this study is consistent with these studies (Smith 50%, Khanna 45%). If MRSA was detectable in a farm it was generally easily detectable by either pig or environmental samples. MRSA was detected in approximately 60% of the samples collected at MRSA-positive farms. A higher level of detection was seen in pigs from MRSA-positive farms, but the results were not conclusive. In fact, in one farm all pigs were negative while MRSA was detectable in the environment. In all farms with both pig and environmental testing MRSA status matched 97.4% (37/38) of the time indicating either method is equally likely to detect MRSA from a positive farm. Environmental dust samples have been used for surveillance purposes in other studies [6], [44] and in practice environmental samples are a more convenient method of collection versus live animals. Although this study was not designed to assess risk factors for MRSA on pork farms, there was a strong relationship between presence of young pigs (<10 weeks of age) and detection of MRSA (OR  = 5.95). Other studies have reported an age-related association with MRSA status with highest prevalence reported in piglets between 6–12 weeks of age [8], [45].

### *spa* types

The findings of many studies investigating MRSA in pork farms have indicated that ST398 is the predominant MLST present. In fact, discovery of an untypeable strain of MRSA in the Netherlands and subsequent investigations linking this strain to ST398 and pork farms initiated the process leading to the term “livestock-associated” MRSA [4], [5], [9], [31], [46], [47]. There were 6 *spa* types observed in this study (t002, t034, t126, t548, t1107, t10065) associated with 3 sequence types (ST5, ST398, ST72). However, non-ST398 *spa* types (t002, t548, t1107) predominated and accounted for 84% of the *spa* types observed and were found on 75% MRSA-positive farms. On the other hand, ST398-associated *spa* types (t034, t10065) accounted for 15% of *spa* types observed and were found on only 3 of 12 MRSA-positive farms. MRSA ST5 has been isolated from backyard-raised pigs in Michigan [48] and MRSA t002 was found in Canadian pigs [7], pigs at agricultural fairs [49], U. S. pork products [50], [51], and recently from Ohio pork farms [52]. This study also documents MRSA ST5 subtypes (t002 or t548) directly from pork farms in the U.S. Other studies indicate that non-ST398 (ST9) MRSA strains can be found in pigs and pig carcasses in Asia [44], [53]–[55]. Thus is appears that LA-MRSA is more diverse than ST398-associated strains and geographic differences exist.

Studies using whole-genome sequence typing have examined differences between livestock- origin and human- origin ST398 isolates [56], [57]. The first study reported that human-associated isolates carried phages that were largely missing from livestock-associated isolates. These phages were associated with innate immunomodulatory genes and considered virulence factors in humans. The authors theorized that during the jump to livestock these genes were lost, antibiotic resistance genes gained, and the resulting strains became less capable of re-infecting humans. The Uhleman study similarly reported differences in mobile genetic elements between human- and livestock- associated ST398 strains, but also reported enhanced adhesion of human isolates to human skin keratinocytes and keratin. Both studies found that genes responsible for PVL toxin production were missing in all livestock-associated ST398 strains. Similarly, in our study all ST398 and non-ST398 isolates lack *lukS-lukF*. Taken together, a picture that appears to be emerging is one of initial transmission of human-associated *S. aureus* strains or subtypes to livestock facilitated by loss of human virulence factors. However once established in livestock, the ability to re-infect humans appears reduced, albeit not totally eliminated. MRSA ST398 is perhaps only one example of this process that may have occurred in other sequence types. A similar scenario was reported to be associated with the introduction of human *S. aureus* ST5 into chickens and broilers and subsequent global dissemination [58]. In that study, Lowder provided evidence that subtypes of ST5 found in poultry had undergone genetic diversification leading to acquisition of avian-specific accessory genes and inactivation of human virulence genes. This study suggests a similar process may have occurred with subtypes of ST5 leading to host-adaptation in swine with as yet only local distribution.

### Antimicrobial resistance patterns

All isolates were resistant to spectinomycin, an aminocyclitol. Spectinomycin resistance in ST398 has been reported [59]–[61], however at lower levels than found here. Resistance to tetracycline derivatives (chlortetracycline, oxytetracycline) overall was quite high (87%). Tetracycline resistance is a common feature of ST398 [24], [62], but was also found here with high frequency in non-ST398 isolates (84%). Aminoglycoside resistance (gentamicin, neomycin) averaged approximately 48% with neomycin resistance much higher than gentamicin. A striking difference in neomycin resistance between non-ST398 (87%) and ST398 (8%) isolates was observed. Macrolide resistance (tilmicosin) was 34% while lincosamide (clindamycin) resistance was just over 46%. As a class, the least resistance was seen with sulfonamides (sulfadimethoxine, trimethoprim/sulfamethoxazole). Fluoroquinolone (enrofloxacin) resistance was 16% and resistance to florfenicol, a phenicol derivative, was nearly 39%. A Belgian study [42] which tested 643 pig MRSA ST398 isolates reported similar resistant rates in comparable drug classes for tetracycline (100%), aminoglycosides (48%), macrolides (56%), and sulfonamides (2%). However, that study found higher resistance with lincosamides (73%), and fluroroquinolones (32%), and lower resistance to the phenicol derivative, chloramphenicol (5%). In this study pleuromutilin resistance (tiamulin) was 22%. Additionally, tiamulin resistance appeared to be associated with clindamycin resistance (12/15), which may indicate presence of *vga*(A) as recently reported in ST398 [63]. There was a wide diversity of resistance phenotypes found in the isolates tested in this study with combined resistant to tetracyclines, neomycin, and spectinomycin seen most commonly particularly in ST5 subtypes. These subtypes were also more likely to be multidrug resistant.

Resistance patterns can be expected to vary based on location, drug approval, and farm level management. Due to study constraints, site-specific antimicrobial use was not recorded. Other limitations in this study include non-random selection of production sites and clustering of sites within production systems. Since the selection of pork production sites that were sampled was based on a request for assistance to the ISU Swine Production Group, presumably health-related problems existed at the farm. Management practices and farm conditions which contribute to health problems may also contribute to the presence of MRSA. Additionally, it is not uncommon for swine course diagnostic investigations to involve multiple pork farms within a common production system. Therefore, use of common practices, equipment, and breeding stock could lead to MRSA contamination of multiple farms and significantly affect the prevalence of particular MRSA strains. Detailed information on the pork farms was withheld in this study.

### Conclusions

The findings from this study support some of the findings from other studies. We found that following short-term exposure to MRSA-positive pork farms MRSA could be detected in students 22% of the time, but this level of exposure did not lead to stable colonization in participants. The prevalence of MRSA in pork farms was 30%, which is lower than results from many prevalence studies in Europe, but similar to results from other studies in North America. One of the surprising findings was the predominance of ST5 subtypes on farms and in students. ST398 subtypes were not detected in any exposed student. It was interesting that some the characteristics of the these non-ST398 isolates resembled ST398 in that none contained the PVL toxin gene but were likely to be tetracycline resistant. However, non-ST398 isolates differed in their resistance profile particularly in regard to a high level of resistance to neomycin and association with multidrug phenotype. Further investigation of these isolates by molecular analysis is needed to determine if these isolates fit the pattern associated with LA-MRSA, but it seems likely that MRSA subtypes from multiple lineages have made the human-to-livestock leap. Whether the impediments to human re-adaptation remain in place is still unknown.
